# The Role of Phlebotomy (Fasd) and Cupping in the Treatment of Epilepsy from Perspective of Persian Medicine

**Published:** 2019-07

**Authors:** Fatemeh EGHBALIAN, Hoorieh MOHAMMADI KENARI, Gholamreza KORDAFSHARI, Mehrdad KARIMI, Akramosadat ATYABI, Laila SHIRBEIGI

**Affiliations:** 1. Research Institute for Islamic and Complementary Medicine, School of Iranian Traditional Medicine, Iran University of Medical Sciences, Tehran, Iran; 2. Department of Traditional Medicine, School of Persian Medicine, Tehran University of Medical Sciences, Tehran, Iran

## Dear Editor-in-Chief

Epilepsy is a prevalent temporary cerebral dysfunction that affects the patient’s quality of life. About 30% of epileptic patients suffer from drug resistance & side effects of chemical drugs (1). Therefore, there is a tendency to use complementary and alternative medicines such as Persian medicine (PM), around the world. PM includes a set of knowledge and skills to identify prevent and eliminate diseases based on the humoral theory. According to humoral theory, there are four fundamental Humors in human body known as blood, yellow bile, black bile and phlegm. One of the most important causes of disease is a qualitative or quantitative imbalance in this humor called Su-e-Mizaj (2).

From the point of view of PM, epilepsy is a chronic central nervous system disease, which occurs, in a sudden and periodic attack. Epilepsy has been divided into 2 types (3):

A- Cerebral epilepsy: The pathogenic substance is produced in the brain and depending on the type of dominant substance is divided into 4 groups:

Bloody cerebral epilepsy (Sare-e-Damavi Demaqi)
Yellow Biliary cerebral epilepsy (Sare-e-Safravi Demaqi)
Phlegmatic cerebral epilepsy (Sare-e- Balqami Demaqi)
Black Biliary cerebral epilepsy (Sare-e- Sodavi Demaqi)

B- Non-cerebral epilepsy:

*The cause of the disease is outside the body:
  - Sting epilepsy (Sare-e-Lasie)
  - Posttraumatic epilepsy
*The cause of disease is in another organ such as stomach and uterus, but presents in brain and the disease occurs due to different channels like connective tissue and nervous connections between two organs (Sare-e-Sherki or participatory) (4,5).

Treatments are divided into two kinds; treatments during attacks to prevent harm to members and treatments before onset of the attacks, to prevent relapse of disease. The PM treatments are based on three principles: lifestyle modification, herbal medication and use of manual practices. In some cases, cupping is recommended on the related organ in attack phase. In latency time, treatment of dystemperament is planned. Nutritional and drug are not efficient for treatment, phlebotomy (Fasd) and cupping, can be useful (5,6).

Fasd is a general method for body cleaning from abnormal humor. In this method, a small incision is made by using a sterile blade on the special veins. Vein selection and amount of blood drawing depend on the patient’s physical strength and the type of the disease (7).

Cupping is a method of local purifying that improves connective tissue status. There are two cupping methods. The first one is dry cupping or “Badkesh”, which no blade is used in this method and blood is not drawn after cupping. The second one is wet cupping (“Hijamat”), which small amount of blood is removed after fine scratches on the skin with a sterile blade and suction (5, 8) (Table 1).

The most important recommendations for patients with epilepsy are saphenous vein phlebotomy or wet cupping on the back of both legs and strong body massage from head to bottom.

**Table 1:** Recommendations places for Cupping & phlebotomy in epilepsy (5,7,9)

***Disease***	***Phlebotomy Veins***	***Wet cupping***	***Dry cupping***
Bloody cerebral epilepsy	Cephalic^1^ Sublingual^2^ Saphenous^3^ Median cubital^4^	Cervical^6^ Intra-shoulder^7^ Legs^8^	Legs Head
Black Biliary cerebral epilepsy	Cephalic Median cubital	Shoulder Legs	
Phlegmatic cerebral epilepsy	Cephalic Median cubital	Shoulder^9^ Legs Cervical Occipital^10^	Legs
whole body Partnership epilepsy	Cephalic Sublingual Saphenous Median cubital	-	-
Gastric epilepsy	Cephalic Median cubital Basilica^5^ Saphenous	-	-
Liver epilepsy	Basilica	-	-
Uterine epilepsy	Saphenous	-	-

Persian medicine terms: 1-Qifal, 2- Zir-e-Zaban, 3-Safen, 4-Akhal, 5- Basliq, 6-Noqreh, 7- Kahel, 8-Saqein, 9-Mancab, 10-Qamah-dovah

Bloodletting should be done based on medical supervision, taking a complete history and a physical examination. In cases such as anemia, pregnancy, obesity, excessive or very cold climatic conditions bloodletting must be avoided because of the risk of weakness and reduced body impotence. However, the bloodletting process may be done with caution in emergencies based on the patient’s condition. It is necessary to inform the patient about treatment steps before and after the bloodletting process and it must be done after obtaining the patient’s consent (10).

In addition to long-lasting effects of phlebotomy or cupping, these remedies are simple and cheap based on Persian medicine. Therefore, considering the low number of research articles, it seems necessary to conduct further clinical trials in these methods to evaluate their effects on different kinds of diseases.
